# Differential Responses of Post-Exercise Recovery of Leg Blood Flow and Oxygen Uptake Kinetics in HFpEF versus HFrEF

**DOI:** 10.1371/journal.pone.0163513

**Published:** 2016-10-04

**Authors:** Richard B. Thompson, Joseph J. Pagano, Kory W. Mathewson, Ian Paterson, Jason R. Dyck, Dalane W. Kitzman, Mark J. Haykowsky

**Affiliations:** 1 Department of Biomedical Engineering, University of Alberta, Edmonton, Canada; 2 Division of Cardiology, Mazankowski Alberta Heart Institute, University of Alberta, Edmonton, Canada; 3 Department of Pediatrics and Pharmacology, University of Alberta, Edmonton, Canada; 4 Cardiology and Geriatrics, Wake Forest School of Medicine, Winston-Salem, North Carolina, United States of America; 5 College of Nursing and Health Innovation, University of Texas at Arlington, Arlington, Texas, United States of America; University of Louisville, UNITED STATES

## Abstract

The goals of the current study were to compare leg blood flow, oxygen extraction and oxygen uptake (VO_2_) after constant load sub-maximal unilateral knee extension (ULKE) exercise in patients with heart failure with reduced ejection fraction (HFrEF) compared to those with preserved ejection fraction (HFpEF). Previously, it has been shown that prolonged whole body VO_2_ recovery kinetics are directly related to disease severity and all-cause mortality in HFrEF patients. To date, no study has simultaneously measured muscle-specific blood flow and oxygen extraction post exercise recovery kinetics in HFrEF or HFpEF patients; therefore it is unknown if muscle VO_2_ recovery kinetics, and more specifically, the recovery kinetics of blood flow and oxygen extraction at the level of the muscle, differ between HF phenotypes. Ten older (68±10yrs) HFrEF (n = 5) and HFpEF (n = 5) patients performed sub-maximal (85% of maximal weight lifted during an incremental test) ULKE exercise for 4 minutes. Femoral venous blood flow and venous O_2_ saturation were measured continuously from the onset of end-exercise, using a novel MRI method, to determine off-kinetics (mean response times, MRT) for leg VO_2_ and its determinants. HFpEF and HFrEF patients had similar end-exercise leg blood flow (1.1±0.6 vs. 1.2±0.6 L/min, p>0.05), venous saturation (42±12 vs. 41±11%, p>0.05) and VO_2_ (0.13±0.08 vs. 0.11±0.05 L/min, p>0.05); however HFrEF had significantly delayed recovery MRT for flow (292±135sec. vs 105±63sec., p = 0.004) and VO_2_ (95±37sec. vs. 47±15sec., p = 0.005) compared to HFpEF. Impaired muscle VO_2_ recovery kinetics following ULKE exercise differentiated HFrEF from HFpEF patients and suggests distinct underlying pathology and potential therapeutic approaches in these populations.

## Introduction

The primary chronic symptom in heart failure patients with reduced or preserved ejection fraction (HFrEF and HFpEF, respectively), even when stable and well compensated, is severe exercise intolerance which is associated with their reduced quality of life [1]. The majority of prior studies that have examined the mechanisms of exercise intolerance in HF have measured hemodynamic and metabolic responses during peak aerobic exercise; however the time course of the change in pulmonary oxygen uptake (pulm VO_2_) in the recovery period after exercise also provides important clinical and prognostic information. Specifically, prolonged pulm VO_2_ recovery kinetics are directly related to disease severity (measured as NYHA class) and all-cause mortality, and inversely related to peak aerobic power in HFrEF patients [2–9]. Recovery kinetics after constant load sub-maximal exercise are also relatively insensitive to exercise intensity [5, 10], which has important practical advantages.

Belardinelli et al. [2] reported that pulm VO_2_ and skeletal muscle oxygenation recovery kinetics (measured with near infrared spectroscopy, NIRS) were significantly delayed in HFrEF patients compared to healthy controls after performing constant-load sub-maximal exercise. The prolonged muscle oxygenation recovery kinetics found in HFrEF patients has been associated with abnormalities in peripheral vascular and/or skeletal muscle function that was associated with delayed recovery of muscle blood flow or impaired skeletal muscle oxygen delivery and utilization following exercise [2, 3, 5, 11–13]. However, the independent contributions of blood flow and oxygen extraction to overall oxygen consumption during recovery following isolated muscle exercise, where the heart is not a major limiting factor as occurs during unilateral knee extension (ULKE) exercise [14], have not been previously been measured in HFrEF and HFpEF patients. The goals of the current study were to compare skeletal muscle blood flow, oxygen extraction and oxygen consumption recovery kinetics following ULKE exercise in HFrEF and HFpEF patients.

## Methods

### Subjects

The subjects for this study included 10 heart failure patients, HFrEF (n = 5) and HFpEF (n = 5), recruited from the Alberta Heart Failure Etiology and Analysis Study [15]. All patients were clinically stable (NYHA class I and II) with no medication change in the past three months. Data acquired using the same exercise challenge and non-invasive imaging methods were also included from healthy younger individuals previously reported from our laboratory to highlight the relatively rapid recovery kinetics for leg VO_2_ and its determinants in health [16]. Informed written consent was obtained from all subjects, and the study was approved by the University of Alberta Health Ethics Research Board.

### Unilateral Knee Extensor Exercise

All subjects performed an incremental exercise test (50 knee extensions/minute) using a custom designed MRI compatible ULKE exercise device [16]. The first 30 seconds consisted of unloaded KE exercise, thereafter 100g of weight was added every 30 seconds until volitional exhaustion or until the subject was unable to adhere to the pre-set cadence.

After a 45-minute rest period, subjects performed KE exercise at 85% of the maximal weight lifted in the incremental exercise test for a duration of 4 minutes at a cadence of 50 knee extensions/minute, inside the MRI scanner. Blood pressure (cuff syphgmomanometer) and arterial oxygen saturation (SaO2, digital pulse oximeter) were measured during exercise. Blood was drawn from all subjects prior to exercise for measurement of hemoglobin (Hgb) and hematocrit (Hct).

### Imaging

**Fig 1:**
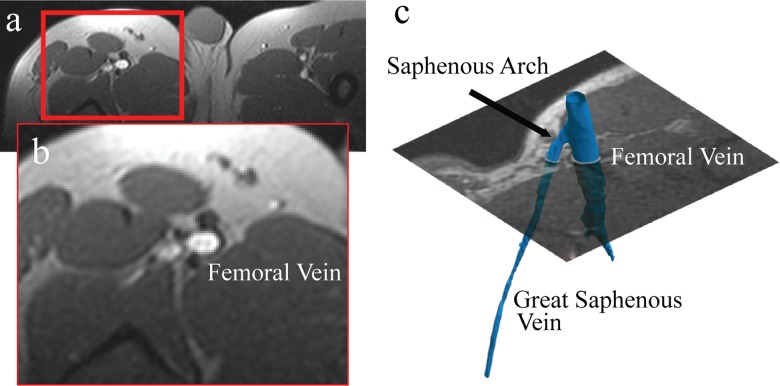
Femoral vein slice prescription. (**a**) Anatomic image showing the slice orientation, perpendicular to the targeted femoral vein, with a close-up view in (**b**). The location of the slice, relative to the femoral vein and great saphenous vein is shown in (**c**), with targeting of the femoral vein prior to the saphenous arch.

Leg (femoral venous) blood flow and O_2_ saturation (SvO_2_) were measured in all subjects, from the onset of end-exercise, using magnetic resonance imaging as previously described [16]. Localizer images were used to prescribe the imaging plane for measurement of blood flow and SvO_2_ perpendicular to the long axis of the femoral vein, proximal to the circumflex and distal to the junction of the greater saphenous vein, as shown in Fig 1. KE exercise was performed in the MRI scanner with the femoral vein landmarked to the center of the bore to ensure imaging could begin at the onset of end-exercise (within 1 second) without patient re-positioning. Blood flow and SvO_2_ image acquisitions were repeated every 5 seconds for 200 seconds. Following exercise studies, additional volumetric images covering the entire quadriceps muscle group were acquired for quantification of muscle mass.

### Data Processing

Femoral venous oxygen saturation was calculated using the known magnetic susceptibility effects of deoxyhemaglobin [16], which gives rise to a directly measurable shift in the magnetic field within the vein lumen, relative to the magnetic field in the surrounding tissue [17, 18]. Femoral venous blood flow was measured using a complex-difference method, as previously described [16, 19]. Flow and oxygen saturation were used to estimate leg muscle VO_2_ using the Fick equation, VO_2_ = Flow * a-vO_2_ diff, where the arterial-venous oxygen difference can be approximated as a-vO_2_ diff = Hgb*1.34*(SaO_2_-SvO_2_), where each gram of hemoglobin carries 1.34 ml of O_2_ and SaO_2_ is the arterial oxygen saturation. The values for leg (femoral vein) blood flow (L/min and L/min/kg), SvO_2_(%), a-vO_2_ diff (mL/100mL) and VO_2_ (L/min and L/min/kg) were calculated at end-exercise (within 1 second of exercise cessation) and continuously, every 5 seconds, for 200 seconds. Recovery kinetics were quantified using the mean response time (MRT), which is defined as the sum of the exponential time constant of decay plus a delay term, from end-exercise to the onset of exponential decay.

Normalization of blood flow and VO_2_ to muscle mass was based on the total quadriceps muscle volume. The quadriceps muscle group was traced on each slice of the thigh volumetric images set and the final volume was multiple by 1.06 g/ml to calculate mass. Expired gas cardiopulmonary VO_2_ peak and MRI-derived cardiac structure and function were measured in a previous study as part of the Alberta Heart Failure Etiology and Analysis Study [15], including left ventricular (LV) end-diastolic and end-systolic volumes (EDV and ESV, respectively), ejection fraction (LVEF), cardiac output and LV mass.

### Statistical Analysis

The t-test for independent samples was utilized and data are presented as mean ± standard deviation. Relationships between variables were assessed by Pearson’s product-moment correlation. Two-way repeated measures analysis of variance (ANOVA) was used to test for mean differences between and within heart failure and control subjects for MRT. *A priori*, *P*<0.05 was considered significant.

## Results

### Subject Characteristics

No significant difference was found between HF groups for age, body surface area, quadriceps muscle mass, resting blood pressure, heart rate, cardiac output, LVM, Hgb, Hct, resting arterial or venous saturation, or peak muscle oxygen uptake (Tables 1 and 2). LV EDV and ESV were significantly larger in HFrEF patients while LVEF and LVM/EDV were significantly reduced in HFrEF patients (Table 1).

**Table 1:** Participant Characteristics.

	HFpEF (n = 5, male)	HFrEF (n = 5, male)
Age, years	67±11	69±9
BSA, m^2^	2.28±0.16	2.01±0.11
SBP, mmHg	142.2±4.5	124.8±29.7
DBP, mmHg	82.6±6.4	75.2±4.8
HR, bpm	77.0±14.4	67.0±19.9
LV EDV, ml	122.6±25.3	213.4±54.7*
LV EDV_,_ ml/m^2^	53.8±9.4	102.8±29.0*
LV ESV, ml	58.8±23.9	130.2±50.8*
LV ESV_,_ ml/m^2^	25.8±10.0	62.4±24.7*
SV, ml	72.6±21.2	83.4±33.1
CO_,_ L/min/m^2^	2.6±1.0	2.8±1.4
LVEF, %	56.6±5.5	36.4±12.0*
LVM, g	155.2±27.5	161.0±26.3
LVM, g/m^2^	68.6±13.2	77.4±13.4
LVM/ LVEDV, g/ml	1.30±0.34	0.77±0.09*
Hgb, g/dl	14.7±2.1	13.9±0.9
Hct, %	0.44±0.06	0.42±0.03
BNP (pg/ml)	117±155	102±117
NT-proBNP (pg/ml)	144±132	212±245
Venous saturation (rest), %	68.1±6.7	57.4±13.5
Arterial saturation (rest), %	96±2	97±2
NYHA Class, n		
I	3	4
II	2	1
History of hypertension,	5	4
Peak VO_2_, ml/kg/min	18.5±5 (n = 4)	18.3±2 (n = 5)
Medications, n		
Diuretics	2	3
ACE	3	3
BB	4	4
CCB	0	1

*p<0.05

ACE, angiotensin-converting enzyme inhibitors; BB, beta-blockers; BSA, body surface area; CCB, calcium channel blockers; CO, cardiac output; EDV, end-diastolic volume; EF, ejection fraction; ESV, end-systolic volume; Hct, hematocrit; Hgb, hemoglobin; LV, left ventricle; LVM, left ventricular mass; NYHA, SV, stroke volume

**Table 2:** MRI parameters obtained at rest and end of sub-maximal unilateral knee extensor exercise.

	HFpEF	HFrEF
Quadriceps mass, kg	1.72±0.36	1.44±0.23
Quadriceps mass indexed to BSA, kg/m^2^	0.76±0.15	0.71±0.09
Femoral Vein Angle, °	21±9	18±5
Weight lifted during continuous exercise, kg	2.0±0.3	2.3±0.9
Work during continuous exercise, Watts	2.3±0.3	2.6±1.0
End-exercise femoral flow, L/min	1.06±0.55	1.15±0.56
End-exercise femoral flow indexed to quadriceps mass, L/min/kg	0.59±0.20	0.82±0.41
End-exercise HR, bpm	95.4±9.1	84.6±19.8
End-exercise SBP, mmHg	157.4±13.0	134.4±36.5
End-exercise DBP, mmHg	94.4±7.2	84.2±8.4
End-exercise SvO_2_, %	41.8±11.8	40.8±10.5
End-exercise SaO_2_,%	95.0±2.0	98.0±2.0
End-exercise AVO_2_ Diff, mL/100mL	10.3±2.7	10.5±2.2
End-exercise muscle VO_2_, L/min	0.13±0.08	0.11±0.05
End-exercise muscle VO_2_ indexed to quadriceps mass, L/min/kg	0.07±0.03	0.08±0.03

AVO_2_ Diff, arterial–venous oxygen content difference; DBP, diastolic blood pressure; HR, heart rate; BSA, body surface area; SaO_2_, arterial oxygen saturation; SvO_2_, venous oxygen saturation; SBP, systolic blood pressure

The two HF groups had similar distributions of NYHA class, all class I or II, similar pulm VO_2_ peak values from their most recent tests (18.5±5.0 ml/kg/min for HFpEF and 18.3±2.0 ml/kg/min for HFrEF patients), and similar use of HF medications as summarized in Table 1.

### Sub-maximal exercise hemodynamics and post exercise muscle VO_2_ recovery kinetics

**Fig 2:**
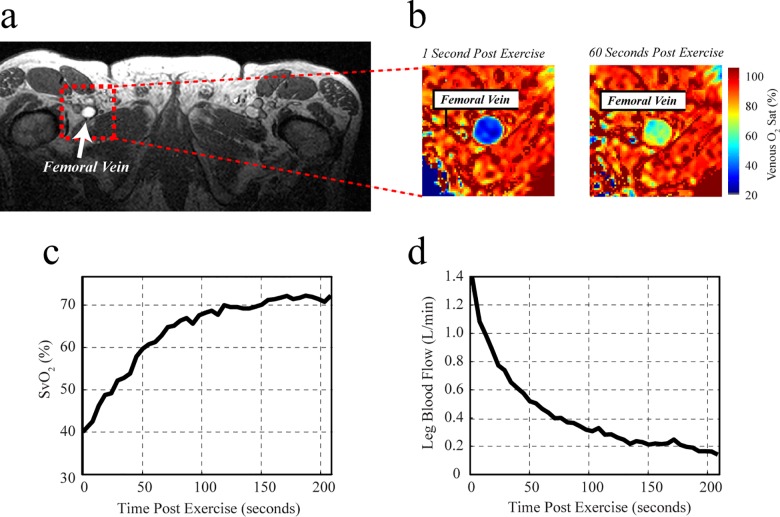
Sample MR images of femoral vein and SvO_2_. (**a**) Anatomic image showing the slice location for blood flow and venous oxygen saturation imaging experiments (the targeted right femoral vein is indicated). Sample venous oxygen saturation (SvO_2_) images at two time points (1 second after end-exercise and 60 seconds after end-exercise) are shown in (**b**), and the corresponding time-course data from this subject for SvO_2_ and blood flow, averaged for the entire vein cross-section, are in (**c**) and (**d**), respectively.

The maximal weight lifted during the ULKE exercise test and thus the weight during sub-maximal (constant-work load) exercise was not significantly different between groups (Table 2), and all subjects completed the 4 minutes of constant work-load exercise at 85% of their maximum weight within the MRI scanner. End-exercise heart rate, blood pressure, femoral blood flow, SvO_2_, a-vO_2_diff, and muscle VO_2_ (absolute or indexed to quadriceps mass) were also not significantly different between HFpEF and HFrEF patients (Table 2). Fig 2A illustrates a typical imaging slice orientation, perpendicular to the targeted femoral vein, and the corresponding venous oxygen saturation images at two sample time points, immediately post-exercise and ~60 seconds post-exercise, in Fig 2B. The post-exercise time course of venous oxygen saturation and blood flow in this subject, averaged over the vein lumen, is shown in Fig 2C and 2D, respectively.

**Fig 3:**
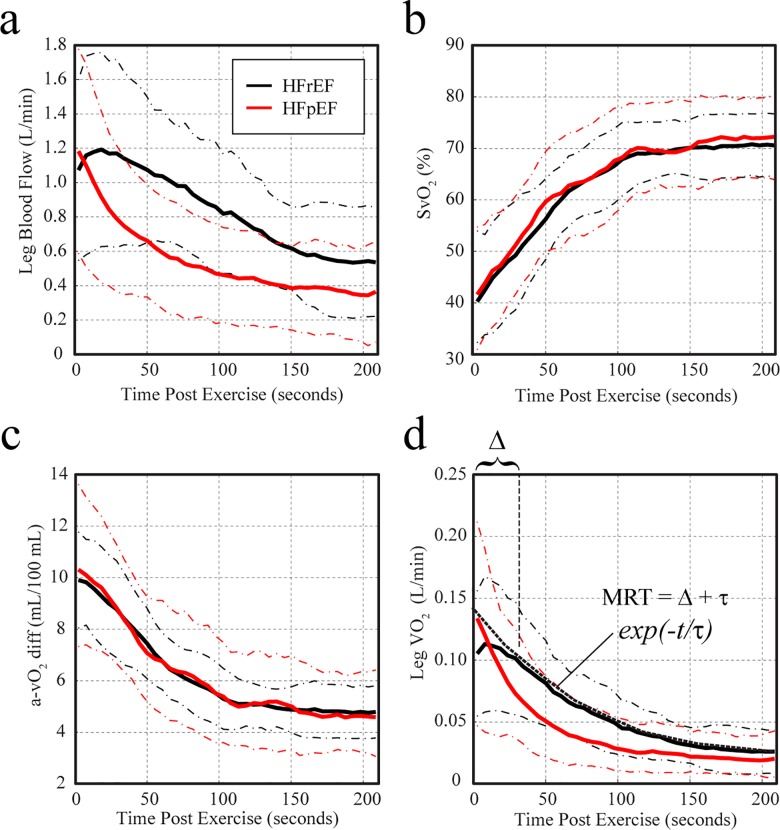
Group average recovery kinetics for blood flow and oxygen extraction and consumption. Average time course data for femoral vein blood flow (**a**), femoral venous oxygen saturation (**b**), a-v O_2_ diff (**c**) and muscle VO_2_ (**d**) are shown for HFrEF (black) and HFpEF (red) groups. The dashed lines show one standard deviation around the mean. The mean response time (MRT) for each curve is defined as the sum of the best-fit exponential recovery plus the delay to the onset of exponential recovery.

**Fig 4:**
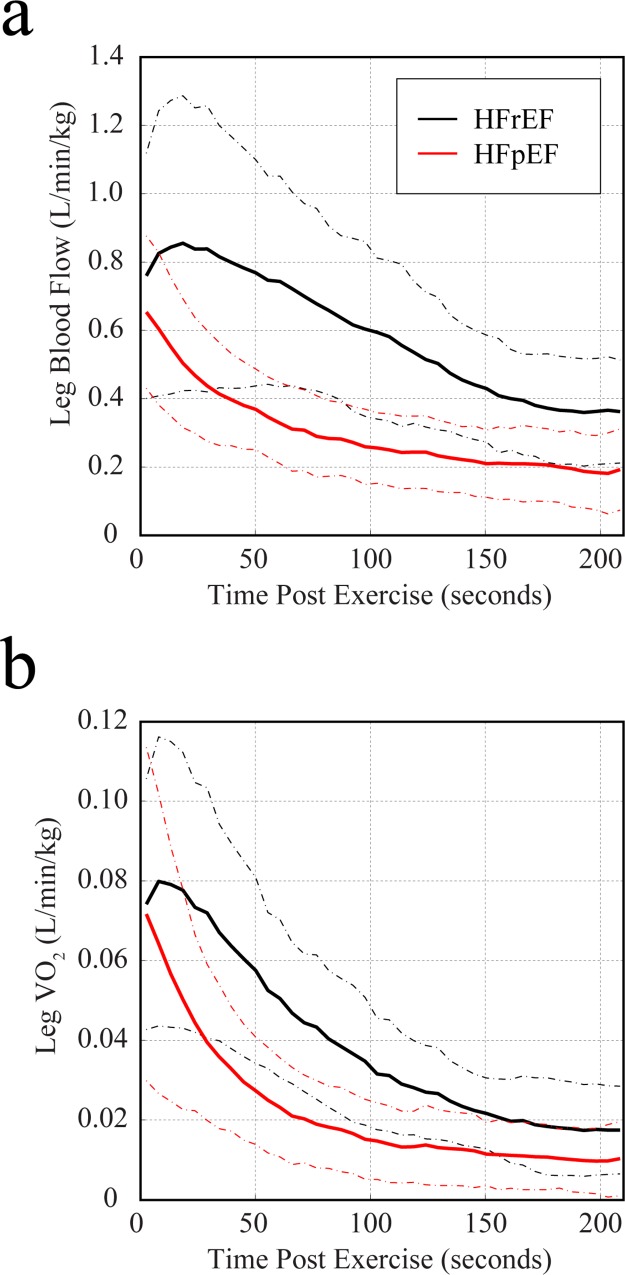
Group average recovery kinetics for blood flow and oxygen consumption (normalized to muscle mass). Average time course data for muscle blood flow (**a**) and muscle VO_2_ (**b**) are shown for HFrEF (black) and HFpEF (red) groups, with normalization of values in each subject to their quadriceps muscle mass. The dashed lines show one standard deviation around the mean.

**Fig 5:**
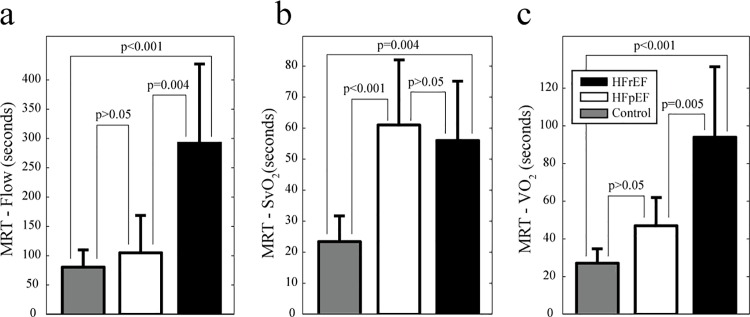
Mean response times. Mean response times (MRT) for (**a**) muscle blood flow, (**b**) SvO_2_ and (**c**) VO_2_ following knee-extension exercise. Control data from a previous study using an identical acquisition protocol [16].

Fig 3 compares the group-averaged recovery kinetics for HFrEF and HFpEF patients, illustrating similar recovery rates for SvO_2_ (Fig 3B) and thus similar a-vO_2_ diff recovery dynamics (Fig 3C), with a peak oxygen extraction of ~60% at end-exercise. However, HFrEF patients had a delayed recovery of muscle VO_2_ (Fig 3D) which is associated with delayed blood flow recovery kinetics (Fig 3A). Normalization of leg blood flow and VO_2_ values to quadriceps muscle mass in each subject (Fig 4A and 4B) further distinguished the recovery dynamics of HFrEF and HFpEF groups. The recovery dynamics for all metrics were evaluated using the mean response time, as defined in Fig 3D. Fig 5 summarizes the post-exercise MRT values for blood flow, SvO_2_ and VO_2_ in HFrEF and HFpEF groups, with an additional comparison to a younger healthy control group, from a previous study using the same imaging methodology [16]. Significant group difference were found for MRT times for leg blood flow, SvO_2_ and VO_2_ (p<0.05). The results of the post-hoc comparisons between groups are shown in Fig 5, where HFrEF patients had significantly delayed recovery of muscle VO_2_ following exercise compared to HFpEF (95±37sec versus 47±15sec, p = 0.004) and healthy controls (95±37sec versus 26±4sec, p < 0.001) as shown in Fig 5C. The delayed recovery of oxygen consumption to baseline in HFrEF patients is associated with the delayed recovery of blood flow in the HFrEF patients as compared to HFpEF (292±135 sec versus 105±63sec, p<0.001)) as shown in Fig 5A.

**Fig 6:**
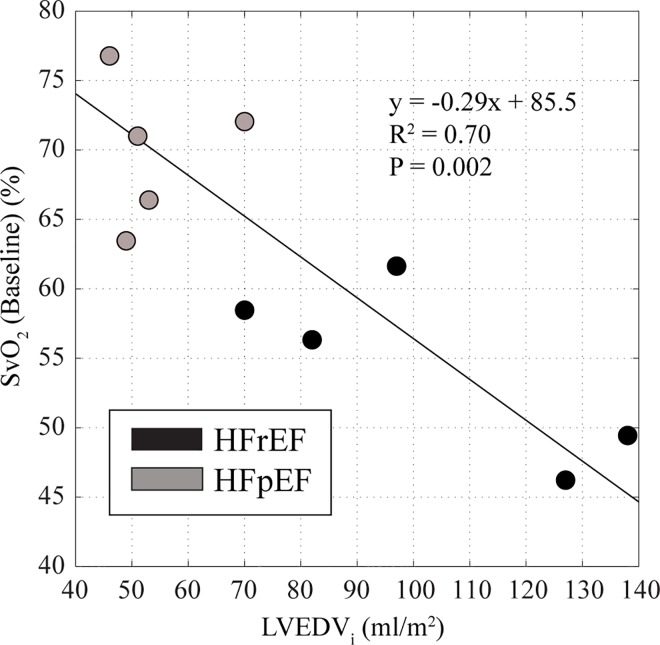
Resting venous oxygen saturation and ventricular remodeling. Relationship between indexed left ventricular end-diastolic volume and resting femoral vein O_2_ saturation (SvO_2_) in heart failure patients.

While HFpEF patients had normal average resting values of 68.1±6.7% for venous oxygen saturation (SvO_2_), similar to previous invasively measured values of 66% [20] and previously reported imaging-derived values of 69% [16], HFrEF patients trended to lower values, 57.4±13.5%, and with a wider range of values. Fig 6 shows the relationship between resting SvO_2_ and indexed EDV (left ventricular end-diastolic volume / body surface area) in all HF subjects, showing a significant relationship between LV dilation and reduced resting SvO_2_ (R^2^ = 0.7, p = 0.003) with a similar relationship between SvO_2_ and LVEF (not shown). However, indexed stoke volume or cardiac output at rest were not related to resting venous oxygen saturation (p>0.05). Finally, peak pulmonary VO_2_ from whole body exercise testing was significantly correlated with the imaging-derived isolated muscle-specific VO_2_ values in HF patients (R^2^ = 0.61, p<0.05).

## Discussion

To our knowledge, this is the first investigation to simultaneously measure and compare muscle blood flow, oxygen extraction and VO_2_ kinetics in the rest-recovery period after sub-maximal ULKE exercise in HFrEF and HFpEF patients. The major new finding of this study is that the recovery MRT of leg blood flow and VO_2_ after constant load sub-maximal ULKE exercise are significantly prolonged in HFrEF versus HFpEF patients.

Currently, only a handful studies have investigated the time course of the change in skeletal muscle oxygenation and/or muscle metabolism in the rest-recovery period after sub-maximal minor muscle mass exercise in HFrEF patients, all of which have shown slower recovery rates in patients. Hanada et al. measured skeletal muscle oxygenated hemoglobin (oxy-Hb) and phosphocreatine (PCr) recovery kinetics using near infrared spectroscopy (NIRS) and phosphorous magnetic resonance spectroscopy (^31^P-MRS) after constant load submaximal calf (plantar flexion) exercise in 13 HFrEF patients (mean age: 58 years, NYHA class II-III) and 15 healthy age-matched subjects [12]. Both PCr and skeletal muscle oxy-Hb recovery rates were significantly slower in HFrEF patients compared to healthy controls (76 vs. 37 seconds, and 48 vs. 30 seconds, respectively). Kemps et al. confirmed that PCr resynthesis and skeletal muscle reoxygenation were delayed in HFrEF patients (n = 13, mean age: 60 years, NYHA class II-III) compared to age and body mass index-matched healthy subjects (n = 8) after constant load submaximal unilateral KE exercise [13] and it was postulated, but not shown, that the delayed recovery after submaximal exercise in HFrEF patients was due to reduced muscle blood flow [13]. Similarly, Bhella et al. measured impaired PCr recovery kinetics in HFpEF patients as compared to healthy controls, but with no comparison to HFrEF patients and no oxygenation or blood flow findings [21].

The current study is the first to directly measure muscle blood flow, oxygen extraction and oxygen consumption in the HFrEF and HFpEF patients. It was found that the delayed leg VO_2_ recovery in HFrEF compared to HFpEF, with significantly longer MRT (95±37 sec versus 47±15 sec) was secondary to the prolonged leg blood flow MRT (292±135 sec versus 105±63 sec), given that SvO_2_ and thus a-vO_2_ diff MRT were not different between groups. Importantly, these differences in recovery between HFrEF and HFpEF subjects occurred with similar group values for end-exercise flow, SvO_2_, a-vO2 diff and VO_2_, along with similar exercise workloads (peak and constant workload) and whole body VO_2_ peak values. Notably, during the first minute of recovery there is an overshoot (increase above the end exercise value) in leg blood flow in HFrEF that is not observed in HFpEF patients or healthy controls (healthy control data previously reported [16]). A similar delay to the time of peak cardiac output following maximal upright exercise has previously been observed in HFrEF patients, which was associated with a reduction in systemic vascular resistance during recovery [3]. Accordingly, our finding of an overshoot in leg blood flow in HFrEF but not HFpEF patients during the first minute of recovery may be due to a relatively lower vascular resistance in HFrEF during this period. Indeed, the magnitude of blood flow at end-exercise was not significantly different between groups while systolic and diastolic blood pressure were higher in HFpEF versus HFrEF patients, which would suggest a lower leg vascular resistance in the HFrEF group during this time. Fig 5 summarizes the distinct recovery dynamics for flow, oxygen extraction and oxygen consumption in the HFrEF, HFpEF and a previously evaluated control group [16]. While both HF groups has similarly impaired recovery of oxygen extraction as compared to controls (HFrEF, 56±19 sec; HFpEF, 62±21 sec; Control, 23±9 sec) (Fig 5B), only the HFrEF group had statistically increased leg VO_2_ MRT (Fig 5C). As shown in Fig 5A, the source of the difference in VO_2_ recovery kinetics in HF groups is the recovery of blood flow, where HFrEF patients has significantly impaired recovery of blood flow to baseline following exercise, as compared to both HFpEF and healthy controls.

In addition to distinguishing groups in the current study, recovery O_2_ kinetics also offer technical advantages over the onset kinetics or peak consumption values. It has previously been shown that recovery kinetics have the highest reproducibility of these parameters [22–24], and also have the advantage of being independent of workload intensity for a wide range of intensities [5, 24, 25]. A coefficient of variation of 6% for reproducibility of muscle VO_2_ recovery kinetics in healthy subjects, using similar methods to those used in the current study, has previously been reported.[16]

Impaired recovery from submaximal exercise in HFrEF patients may ultimately impede their ability to perform repeated bouts of high intensity exercise, for which there is increasing interest as a heart failure therapy [26, 27]. Typical high intensity training protocols, with similar paradigms used in this study (i.e. 85% of maximal effort, 4 minute duration with 3 minutes of recovery) [26, 27], may not offer sufficient recovery time for those with impaired recovery to recuperate energy stores in peripheral muscles.

Finally, the significant negative linear relationship between resting SvO_2_ values and the severity of LV dilation (Fig 6) suggests that fundamental differences in resting skeletal muscle flow and oxygen extraction distinguish HFrEF and HFpEF patients, potentially as a consequence of reduced skeletal muscle blood flow in the HFrEF group at rest that is related to the extent of their LV remodeling. Importantly, resting stroke volume and cardiac output were similar between the two HF groups, and were not significantly related to resting SvO_2_, thus increased muscle oxygen extraction at rest does not appear directly related to resting heart function. It has previously been shown that impaired blood flow to the lower extremities in HFrEF patients with submaximal exercise, with comparison to healthy controls, is independent of their cardiac output and flow in the descending aorta, suggesting an intrinsic peripheral mechanism [28].

The current study has a number of limitations. First, the small number of subjects evaluated limits the generalizability of the findings. Only male subjects were evaluated, and thus the common older female HFpEF phenotype was not represented. Further studies in larger cohorts including age-matched and gender-matched healthy controls are need. Second, while flow and oxygen extraction were measured simultaneously, from which oxygen consumption was calculated, no direct information regarding metabolism (e.g. ^31^P spectroscopy) was acquired, and thus the relationship between the currently reported recovery dynamics and those for inorganic phosphate, adenosine triphosphate (ATP) and phosphocreatine are unknown. Third, the MRI method used in the current study cannot acquire data during exercise, and thus the kinetics of O_2_ uptake during exercise cannot be measured. Fourth, previously published recovery MRT results from a healthy control group [16] were included in Fig 5. The healthy subjects were younger (31±6 years) than the HF patients in the current study, and all performed the same absolute constant workload of 5 Watts, although, using the same exercise device and imaging protocol used in the current study. Given that recovery kinetics have been shown to be largely independent of the workload for sub-maximal exercise (for VO_2_ and ^31^P) [5, 25], it was determined that the MRT data from the younger healthy controls was relevant for comparison with the HF data in the current study. Furthermore, it has been shown that healthy aging does not have a significant effect on the metabolic response to exercise, based on the times to resynthesize adenosine triphosphate following exercise.[29]

In conclusion, impaired recovery of muscle VO_2_ kinetics following isolated muscle exercise differentiated HFrEF from HFpEF patients. While larger studies are necessary to establish the functional and prognostic implications of isolated muscle VO_2_ MRT across the HF continuum (comprising several distinct phenotypes), these findings suggest that distinct mechanisms in the periphery may underlie the impaired muscle oxygen delivery and utilization in patients with chronic HFrEF vs HFpEF, with potentially distinct optimal therapeutic approaches.
